# Moxifloxacin concentration correlate with QTc interval in rifampicin-resistant tuberculosis patients on shorter treatment regimens

**DOI:** 10.1016/j.jctube.2022.100320

**Published:** 2022-06-06

**Authors:** Tutik Kusmiati, Ni Made Mertaniasih, Johanes Nugroho Eko Putranto, Budi Suprapti, Nadya Luthfah, Soedarsono Soedarsono, Winariani Koesoemoprodjo, Aryani Prawita Sari

**Affiliations:** a Doctoral Program of Medical Science, Faculty of Medicine, Universitas Airlangga, Indonesia; b Department of Pulmonology and Respiratory Medicine, Faculty of Medicine, Universitas Airlangga, Surabaya, East Java, Indonesia; c Department of Medical Microbiology, Faculty of Medicine, Universitas Airlangga, Surabaya, East Java, Indonesia; d Laboratory of Tuberculosis, Institute of Tropical Disease, Universitas Airlangga. Surabaya, Indonesia; e Department of Cardiology and Vascular Medicine, Faculty of Medicine, Universitas Airlangga, Surabaya, East Java, Indonesia; f Department of Clinical Pharmacy, Faculty of Pharmacy, Universitas Airlangga, Surabaya, East Java, Indonesia

**Keywords:** Drug-resistant tuberculosis, Shorter treatment regimen, Moxifloxacin concentration, QTc interval

## Abstract

**Background:**

Drug-resistant tuberculosis (DR-TB) continues to be a global threat. Moxifloxacin is one of the components of the shorter treatment regimen which is suspected to increase the risk of QT prolongation, although it is also likely to be the most effective against DR-TB. A study to evaluate the correlation between the concentration of moxifloxacin and QTc interval in RR-TB patients who received shorter regimens is needed.

**Methods:**

This was an observational study in 2 groups of RR-TB patients on shorter treatment regimens (intensive phase and continuation phase), contain moxifloxacin with body weight-adjusted dose. Blood samples were collected at 2 h after taking the 48th-hour dose and 1 h before taking the 72nd-hour dose.

**Results:**

Forty-five RR-TB patients were included in this study. At 2 h after taking the 48th-hour dose, the mean of QTc interval in intensive phase and continuation phase was 444.38 ms vs. 467.94 ms, p = 0.026, while mean of moxifloxacin concentration in intensive phase and continuation phase was 4.3 µg/mL vs. 4.61 µg/mL, p = 0.686). At 1 h before taking the 72nd-hour dose, both moxifloxacin concentration and QTc interval in intensive phase and continuation showed no significant difference with p-value of 0.610 and 0.325, respectively. At 2 h after taking the 48th-dose, moxifloxacin concentration did not correlate with QTc interval, both in intensive phase (p = 0.576) and in continuation phase (p = 0.691). At 1 h before taking the 72nd-hour dose, moxifloxacin concentration also did not correlate with QTc interval in intensive phase (p = 0.531) and continuation phase (p = 0.209).

**Conclusions:**

Our study found that moxifloxacin concentration did not correlate with QTc interval, which indicates the safe use of moxifloxacin on QTc interval. In addition to close monitoring of QTc interval, the clinicians should also consider other variables which potentially increase risk for QTc prolongation in DR-TB patients who received shorter treatment regimens.

## 1 Introduction

Drug-resistant tuberculosis (DR-TB) continues to be a threat and the barrier for global TB elimination efforts caused 465,000 cases in 2020. Indonesia is rank 5th for countries with high multidrug-resistant/ rifampicin-resistant tuberculosis (MDR/RR-TB) burden in the world with 24,000 cases. Globally, the latest data show treatment success rates of 57% for MDR-TB/rifampicin-resistant TB (RR-TB). The success rate for MDR/RR-TB in Indonesia is < 50% due to the high rate of deaths and loss to follow-up [1]. The increase in MDR-TB has further highlighted the need to improve TB treatment [2].

The treatment of MDR/RR TB using a different combination of 2nd line drugs, usually for 18 months is associated with high cost, greater incidence of adverse reactions, and a high rate of loss to follow-up [3]. A shorter MDR/RR-TB regimen for 9–12 months has been promoted by the WHO in 2016 and becomes a priority for certain MDR/RR-TB patients with eligible criteria. This shorter regimen is also expected to increase the success rate of MDR/RR-TB treatment [4]. Despite the shorter duration of this regimen, the adverse effect remains happened and results in treatment interruption, reduce treatment adherence, and associated with morbidity and mortality [4], [5], [6].

Interval QT prolongation is one of the most common adverse effects in MDR/RR-TB patients who received the shorter regimens. A fluoroquinolone, moxifloxacin is one of the core components of the shorter MDR/RR-TB treatment regimen which has been known to carry a risk of QT prolongation, although it is also likely to be the most effective against MDR-TB [4], [7]. Interval QT prolongation (an abnormality on electrocardiography) is a serious adverse effect that favors the development of cardiac arrhythmias, including Torsade de Pointes (TdP), and leads to sudden cardiac death [7], [8].

According to the national program, the use of moxifloxacin in the shorter regimens is based on body weight. Moxifloxacin is given at 400 mg, 600 mg, and 800 mg. However, the safety and efficacy of the recommended dose are still debated. A study reported 7% of MDR/RR-TB patients switched their regimens from shorter regimen to individual regimens due to the presence of prolonged QT. Moxifloxacin was suggested to cause prolonged QT [3]. The QT interval prolongation of moxifloxacin 800 mg was only slightly greater than moxifloxacin 400 mg [9]. A previous study reported the incidence of ΔQTc > 30 ms and ΔQTc > 60 ms in 21/98 (21.4%) and 10/98 (10.2%) of DR-TB patients who received shorter regimens, respectively. There is no significant difference in the incidence of prolonged ΔQTc in 400, 600, and 800 mg of moxifloxacin dose, and no significant correlation between moxifloxacin dose and ΔQTc [10]. Other studies reported the safety on QT interval of moxifloxacin 400 mg and 600 mg [11], [12].

Low concentrations of anti-TB drugs may be associated with poor treatment outcomes as well as high concentrations of these drugs may cause intolerance and toxic effects [13], [14]. An appropriate dose of moxifloxacin was important to achieve effective treatment without serious adverse effects. This study was conducted to evaluate the correlation between the concentration of moxifloxacin and QTc interval in RR-TB patients who received shorter regimens.

## 2 Methods

### 2.1 Study design and subjects

This was an observational analytic study with a time-series design from September 2019 to February 2020 in Dr. Soetomo Hospital Surabaya, which is the center of East Indonesia TB referral hospital. Consecutive sampling was used in this study. Study subjects were RR pulmonary TB patients based on the GeneXpert examinations who meet inclusion and exclusion criteria. RR-TB patients with age 18 to 65 years old who will start the intensive phase and who are on the continuation phase of shorter treatment regimens were included in this study. RR-TB patients with baseline QTc > 500 ms, potassium < 3.5 mmol/L, magnesium < 1.7 mmol/L, calcium < 8.5 mmol/L, creatinine clearance < 30 cc/m, aspartate aminotransferase - alanine aminotransferase (AST-ALT) > 5x upper limit normal (ULN), body mass index (BMI) < 18 kg/m^2^, on anti-arrhythmia therapy, anti-depressant therapy, with bradycardia, anti-fungal treatment (azoles), erythromycin therapy, and phenytoin therapy were excluded from this study. These exclusion criteria are the risk factors of QT interval prolongation [15], [16], [17]. Therefore, the results of this study will not be affected by these factors.

### 2.2 Operational definition

Rifampicin-resistant tuberculosis (RR-TB) was defined as the results of *Mycobacterium tuberculosis* detected with rifampicin resistance based on GeneXpert MTB/RIF [18]. RR-TB patients in intensive phase were defined as RR-TB patients who are eligible to receive shorter regimens and will start intensive phase of treatment. RR-TB patients in continuation phase were defined as RR-TB patients who have completed the intensive phase (4–6 months), i.e. those who have sputum smear conversion after the 4th, 5th, or 6th month. Shorter regimens were as recommended by the WHO in 2016 and the national program in 2019, consisted of 4–6 Km – Mfx – Eto(Pro) – H^High Dose^ – Cfz – E – Z / 5 Mfx – Cfz – E – Z for 9–11 months [4], [19]. Electrocardiography (ECG) was defined as a 12-lead surface heart recording using an ECG machine. The QT interval is that portion of the ECG that begins at the start of the QRS complex and ends at the termination of the T wave. The QTc referred to the corrected QT interval using the Fredericia formula [15]. The changes of QTc (ΔQTc) referred to the difference between the QT interval at baseline and the QT interval at 2 h after taking the 48th-hour dose, and 1 h before taking the 72nd-hour dose.

### 2.3 Concentration of moxifloxacin

All subjects received standardized shorter regimens as recommended by the WHO in 2016 and the national program in 2019, consisted of 4–6 Km – Mfx – Eto(Pro) – H^High Dose^ – Cfz – E – Z / 5 Mfx – Cfz – E – Z for 9–11 months [4], [19]. Moxifloxacin of 400 mg/tablet (Avelox®, Bayer Health Care) was used and subjects received one of three (400 mg or 600 mg or 800 mg of moxifloxacin). Blood samples were collected at 2 h after taking the 48th-hour dose and 1 h before taking the 72nd-hour dose. Blood samples were taken from RR-TB patients and put into heparin tubes. Blood samples were centrifuged and the plasma was stored in the deep freezer with a temperature of −80 °C). The moxifloxacin concentration was measured by a validated method using High-Performance Liquid Chromatography (HPLC). The separation of moxifloxacin from the plasma matrix using protein precipitation, followed by measurements using the Waters HPLC Alliance e2695 with a detector of Waters 2998 Photodiode Array (PDA). 240 µl of acetonitrile solution (100%) was added to the 200 µl of plasma sample. The sample was then vortexed for 1 min and centrifuged at a speed of 10,000*g* for 5 min. A total of 200 µl of supernatant was put into the vial and injected into the HPLC with an injection volume of 10 µl. Separation using a Sunfire™ C18 column (4.6 × 100 mm, 5 µm; Waters, Ireland). The mobile phase consisted of 0.4% TEA in aquabides with a pH of ± 3 and 100% of acetonitrile (75%:25% (v/v)). The flow rate is 1 ml/min and the PDA detector was set at a wavelength of 296 nm. Accuracy for standard concentration curves is between 95.5% and 103.4%, depends on the standard concentration level. The coefficient of variation for intra- and inter-assay was<7.2% for the range from 0.204 to 10,200µg / mL. The lowest limit value which can be quantified was 0.204 µg/mL.

### 2.4 QTc interval measurement

QTc interval was measured using ECG machine merc BLT E30 (Guangdong Biolight Meditech, Germany, 2017). A lead of II or V5-V6 on ECG was selected to be read. QT interval from QRS complex up to the end of T-wave was measured. The ECG waves are recorded on special graph paper that is divided into 1 mm^2^, paper speed on ECG was 25 mm/s, therefore each 1 small square corresponds to 0.04 s (40 ms). QT interval was 0.04 s multiplied with the number of small squares of QRS complex up to the end of T-wave. The QT interval of at least three successive beats was measured, and the beat with the maximum interval was taken. If the rhythm is irregular, the average QT interval of 3–5 beats was taken. RR interval (area between two consecutive Rs of the QRS complex in the ECG rhythm strip) was 0.04 s multiplied with the number of small squares of R to R. Heart rate (HR) was 1500 divided by the amount of RR small squares. QTc was measured using the Fredericia formula according to the HR of subjects [15]. QTc interval was measured at 2 h after taking the 48th-hour dose, and 1 h before taking the 72nd-hour dose.

### 2.5 Data analysis and ethical statement

The data obtained in this study were analyzed using Pearson or Spearman-rho method in SPSS 21.0 (IBM Corp., Armonk, NY, USA). Statistical results were considered significant if the p-value was < 0.05. This study was approved by the ethics committee of Dr. Soetomo Hospital with ethical clearance number 1444/KEPK/VIII/2019 August 23rd, 2019.

## 3 Results

A total of 45 RR-TB patients were screened. 29/45 (64%) RR-TB patients are in intensive phase and 16/45 (36%) RR-TB patients are in continuation phase. The administration of moxifloxacin in this study is based on body weight. Subjects in this present study were patients who received moxifloxacin dose 600 mg and 800 mg because patients who received moxifloxacin 400 mg were not found in this study. Table 1 shows the characteristics of RR-TB patients in intensive phase and continuation phase. Potassium, calcium, and magnesium levels decreased significantly in continuation phase with p-value of 0.019, 0.001, and 0.003, respectively.

**Table 1:** Characteristics of Study Subjects.

Characteristics	RR-TB on Start of Intensive Phase (N = 29)	RR-TB on Start of Continuation Phase (N = 16)	P-value
Age (years)*	37 (18–62)	44 (19–56)	0.569
Sex**			0.673
Women	13	9	
Men	16	7	
BMI (m/kg^2^)*	20.4 (18.03–28.65)	19.06 (18.26–27.68)	0.530
Diabetes mellitus**	14	5	0.429
Albumin***	3.43 ± 0.28	3.65 ± 0.14	0.002
Sodium (mmol/l)***	139.06 ± 4.09	140.7 ± 5.87	0.266
Potassium (mmol/l)***	4.3 ± 0.45	3.96 ± 0.4	0.019
Calcium mg/dl)***	9.03 ± 0.46	8.67 ± 0.2	0.001
Magnesium (mg/dl)*	2.1 (1.8–2.3)	1.9 (1.8–2.2)	0.003
Moxifloxacin **			0.727
600	17	11	
800	12	5	
QTc Baseline(ms)***	417.28 ± 31.2	455.94 ± 16.6	<0.001
Moxy Conc (48 + 2) (µg/mL)***	4.3 ± 2.32	4.61 ± 2.54	0.686
QTc 48 + 2 (ms)***	444.38 ± 31.25	467.94 ± 35.7	0.026
ΔQTc (48 + 2)-Baseline (ms)*	20 (( −1 7) – (81))	2.5 (( −4 4) – (1 1 5))	0.036
Moxy Conc (72–1) (µg/mL)*	1.01 (0.01 – 3.27)	0.91 (0.01 – 1.61)	0.610
QTc 72–1 (ms)*	448 (386–518)	447 (428–524)	0.325
ΔQTc (48 + 2) - (72–1) (ms)*	0 (( −7 5) – (60))	7.5 (( −7 7) – (52))	0.122

* Median (min–max) using Mann-Whitney Test; ** Chi-square; ***Mean ± Standard Deviation using T-test; BMI = Body Mass Index.

As shown in Table 1, at 2 h after taking the 48th-hour dose, mean of QTc interval in intensive phase and continuation phase showed a significant difference (444.38 ms vs. 467.94 ms, p = 0.026). Mean of moxifloxacin concentration in continuation phase was also higher than intensive phase, although it was not significant (p-value of 0.686). At 1 h before taking the 72nd-hour dose, both moxifloxacin concentration and QTc interval in intensive phase and continuation showed no significant difference with p-value of 0.610 and 0.325, respectively.

Table 2 below showed that there was no correlation between moxifloxacin concentration and QTc interval at 2 h after taking the 48th-hour dose, both in intensive phase and continuation phase with p-value of 0.576 and 0.691, respectively. The change of QTc interval from baseline was also not correlated with moxifloxacin concentration with p-value of 0.415 in intensive phase and 0.353 in continuation phase, respectively. At 1 h before the 72nd^-^hour dose, moxifloxacin concentration also did not correlate with the QTc interval (p-value of 0.531 in intensive phase and 0.209 in continuation phase), also did not affect the change of QTc interval (p-value of 0.813 in intensive phase and 0.464 in continuation phase).

**Table 2:** Correlation Analysis at 2 Hours after the 48th Dose and at 1 Hour before the 72nd- Hour Dose.

			Moxi Conc at 48 + 2			Moxi Conc at 72–1
**Intensive phase**	**QTc at 48 + 2**	R	−0.108	**QTc at 72**–**1**	R	0.121
		P	0.576		P	0.531
	Δ**QTc ((48 + 2) – Baseline))**	R	−0.157	Δ**QTc ((48 + 2) – (72**–**1))**	R	−0.046
		P	0.415		P	0.813
**Continuation phase**	**QTc at 48 + 2**	R	0.108	**QTc at 72**–**1**	R	−0.332
		P	0.691		P	0.209
	Δ**QTc ((48 + 2) – Baseline))**	R	0.249	Δ**QTc ((48 + 2) – (72**–**1))**	R	−0.197
		P	0.353		P	0.464

Correlation Analysis using Pearson or Spearman-rho Test; R: Correlation Coefficient; P: Sig. (2-tailed).

**Fig. 1:**
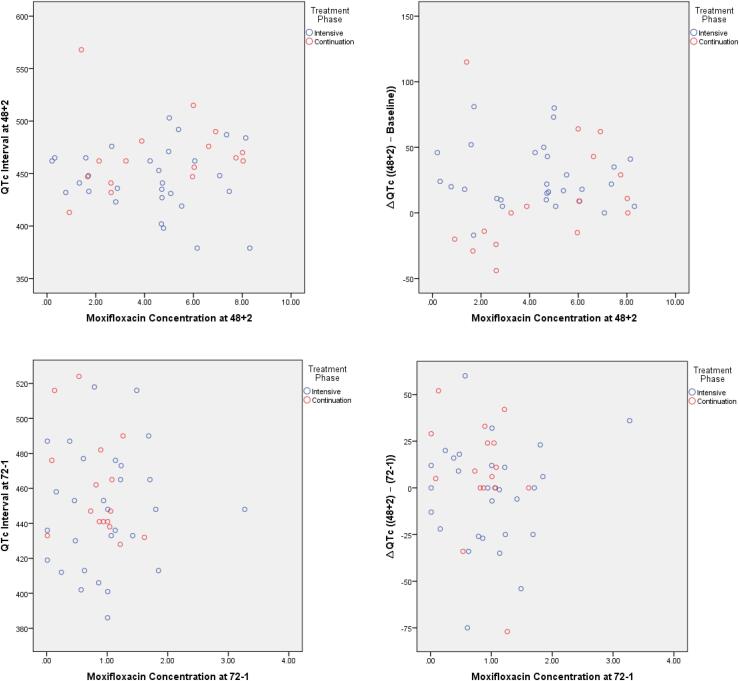
Scatter Plot of Moxifloxacin Concentration (µg/mL) and QTc Interval (ms). 48 + 2 = 2 Hours after Taking the 48th Hour Dose; 72–1 = 1 Hour before Taking the 72nd Hour Dose.

Our study found that there was no correlation between moxifloxacin concentration and QTc interval. Fig. 1 below showed that moxifloxacin concentration and QTc interval at 2 h after taking the 48th-hour dose and at 1 h before taking the 72nd-hour dose) did not form a specific pattern, indicated that there is no correlation between moxifloxacin concentration and QTc interval.

## 4 Discussion

Moxifloxacin is an 8-methoxyquinolone (fluoroquinolone) antimicrobial drug used for treatment of respiratory infections. It is the most commonly used active control in thorough QT studies (TQTS) because it reliably prolongs the QT interval [20], [21], [22]. A single oral dose of 400 mg MX is often used as a positive control in TQT studies [20]. Moxifloxacin is a *repurposed drug* in DR-TB treatment [22]. Fluoroquinolones, especially moxifloxacin is considered as a QT-prolonging drug and as a component in a shorter regimen to treat eligible RR-TB patients [4]. Chen *et al.* reported a significant prolongation of mean QTc at all time points except 0.5 h post-dose after orally administered moxifloxacin. The peak effect on *the Fridericia QT correction* (QTcF) was 9.35 ms at 3 h post-dose. It was suggested that a 2.084 ms increase in the QTc interval for every 1000 ng/mL increase in plasma concentration of moxifloxacin [8].

The current reference dose of moxifloxacin based on body weight seems to be safe, as moxifloxacin concentration in plasma did not correlate with QTc interval in our study (Table 2). Moxifloxacin is generally well tolerated, although moxifloxacin has been shown to prolong the QT interval of the electrocardiogram in some patients by reversible and dose-dependent but weak blockage of the hERG potassium channels. Higher doses (600–800 mg) suggest that these may be safe when carefully monitored [23]. Kusmiati *et al.* also reported that moxifloxacin at the dosage of 400, 600, or 800 mg does not correlate with the QTc interval (p-value of 0.565) [24]. Hong *et al.* reported that the effect by 800 mg was only slightly greater than that of 400 mg, indicated that the QTc interval prolongation effect of moxifloxacin does not double by doubling the dose from 400 to 800 mg [9]. Although fluoroquinolones are known to prolong the QT interval, however, the prolongation is considered minimal or moderate for moxifloxacin [25].

In this study, we have excluded patients with low levels of potassium and calcium to minimize their effects on QTc interval, therefore, potassium and calcium did not correlate with QTc interval (Table 2). As has been known that the QT interval indicates the duration of action potential (AP) in ventricles, which represents the sum of ventricular depolarization and repolarization. AP is caused by the transmembrane flow of ions, including inward depolarizing currents mainly through sodium and calcium channels, and outward repolarizing currents mainly through potassium channels. Six sequentially activated currents are fundamentally involved: the sodium current (INa), the transient outward current (Ito), the L(long-lasting)-type calcium current (ICaL), the rapid component of the delayed rectifier potassium current (IKr), the slow component of the delayed rectifier potassium current (IKs), and the inward rectifier potassium current (IK1) [16]. The majority of the delayed cardiac repolarisations observed in the clinic are believed to be mediated via inhibition of the cardiac potassium ion channel (IKr) which is encoded by the human ether a-go-go gene (hERG) [26]. Most cases of drug-induced long QT result from an action of the drugs on the ion channel proteins encoded by the hERG gene that is responsible for the IKr repolarizing current. Drug-induced QT prolongation is commonly achieved by directly blocking the hERG channel. Fluoroquinolones inhibit Ikr delaying membrane repolarization [27].

Moxifloxacin, a fluoroquinolone antibiotic is known as second-line anti-TB drugs in the medical field, and is critical in DR‑TB treatment [11]. Our study indicates that the use of moxifloxacin may be safe, even with 800 mg. A dose of 800 mg·per day could result in higher bactericidal activity and an improved outcome [22]. Besides, moxifloxacin is also likely to be the most effective against MDR-TB [7], as known that the mechanism of fluoroquinolone antibiotics is mainly the inhibition of the activity of DNA gyrase, and thus the destruction of the replication and transcription of DNA in *Mycobacterium tuberculosis*, which further destroys the genetic material in the cells, leading to the death of *Mycobacterium tuberculosis* [11].

According to Table 1, albumin level was found higher in continuation phase. Sanchez *et al.* reported that albumin levels increased quickly by week 4 of treatment [28]. Albumin concentration decreases as increasing disease severity in chronic infection including TB [29]. Wu *et al.* reported that concentrations of serum albumin were significantly lower in patients with an abnormal QTc interval and were associated with QTc prolongation. Hypoalbuminemia may be a marker of comorbidity burden, a low serum albumin level may reflects an inflammatory burden leading to heart failure [30]. A low albumin level is associated with inflammation, and impaired synthetic function of liver and this has been linked to diastolic dysfunction in humans which indicate the association between inflammation and ventricular arrhythmias [31].

Fig. 1 showed that moxifloxacin concentration and QTc interval did not form a certain pattern, strengthen the results in Table 1, Table 2, that there was no correlation between moxifloxacin and QTc interval. The moxifloxacin concentration also did not affect the change of QTc interval (ΔQTc). An animal study reported different results, moxifloxacin induced a dose-dependent increase in QTc. A maximum increase of 28 ms was observed following the administration of 90 mg/kg moxifloxacin [26].

Our study revealed no correlation between moxifloxacin concentration and QTc interval, suggested the safety use of moxifloxacin, either in 600 or 800 mg. Nachimutu *et al*. stated that the rest of the fluoroquinolones are relatively safe but caution should be applied if there are any underlying risk factors or with co-administration of QT-prolonging drugs [17]. Wang *et al.* also concluded that short‑term treatment with a high dose of moxifloxacin (0.6 g/day for 6 months and 0.4 g/day for 9 months) is effective for MDR-TB, and its advantages are a reduction in the incidence of drug-associated adverse reactions and a lack of drug resistance [11]. Kusmiati *et al.* reported that the QTc interval at baseline correlated significantly with the QTc prolongation (p < 0.001) [24], while Hong *et al.* stated that Koreans appeared to be more sensitive to moxifloxacin-induced QT prolongation than Caucasians [9]. As fluoroquinolones are known to prolong the QT interval, the WHO guideline recommended cardiac monitoring when using drugs that prolong the QT interval [25].

## 5 Conclusions

Moxifloxacin concentration in plasma (either using the dose of 600 or 800 mg) did not correlate with QTc interval. In addition to close monitoring of QTc interval in DR-TB patients, the clinicians should also consider other variables which potentially increase risk for QTc prolongation in DR-TB patients who received shorter treatment regimens.

## Ethical Statement

The authors attest that this clinical investigation was determined to the Institutional Review Board/Ethics Committee review, and the corresponding protocol/approval number is 1444/KEPK/VIII/2019 and has been approved on August 23rd, 2019. We also certify that we have not plagiarized the contents in this submission and have done a Plagiarism Check. Written informed consent was obtained from all participants.

## Declaration of Competing Interest

The authors declare that they have no known competing financial interests or personal relationships that could have appeared to influence the work reported in this paper.
